# The difference in rotation angle of the distal endotracheal tube through nasal approach

**DOI:** 10.1186/s12871-023-02225-7

**Published:** 2023-08-11

**Authors:** Masanori Tsukamoto, Maho Goto, Takashi Hitosugi, Takeshi Yokoyama

**Affiliations:** 1 Department of Dental Anesthesiology, Graduate School of Medical and Dental Sciences, Kagoshima University, Kagoshima, Japan; 2 Department of Dental Anesthesiology, Graduate School of Dental Science, Kyushu University, Fukuoka, Japan; 3 Department of Dental Anesthesiology, Faculty of Dental Science, Kyushu University, Fukuoka, Japan

**Keywords:** Fiberscope, Rotation, Nasotracheal intubation

## Abstract

**Background:**

Nasal intubation using a fiberoptic scope is a useful technique. In clinical practice, we have experienced difficulty in advancing the endotracheal tube (ETT) over the fiberoptic scope because of resistance to the passage of the ETT against rotation in the nasal cavity, when it gets hung up on structures of the laryngeal inlet. Several maneuvers have been proposed to overcome this difficulty. The gap between the tip of the ETT and the fiberoptic scope can be reduced using a thicker fiberoptic scope and a thinner ETT. Moreover, simultaneous rotation of the fiberoptic scope and ETT could lead to successful intubation by reducing impingement on the ETT. However, the discrepancy between these rotation angles is unclear. This observational prospective study aimed to investigate the discrepancy in the rotation angle between the ETT and fiberoptic scope during nasal intubation.

**Methods:**

The patients (aged 20–80 years) who underwent nasal intubation for oral and maxillofacial surgery participated in three sizes of preformed nasal ETT and were intubated using a fiberoptic scope. They were divided into three groups; the ETT internal diameter (ID) 6.5 mm (6.5 group), ID 7.0 mm (7.0 group), and ID 7.5 mm (7.5 group). The ETT was then inserted through the nasal cavity into the pharynx. After the fiberoptic scope was advanced through the ETT above the glottis, simultaneous rotation by both the proximal end of the fiberoptic scope and ETT was performed in 90° and 180° in both right (clockwise) and left (counterclockwise) directions, and the rotation angle at the distal end of the ETT was monitored using a video laryngoscope (Pentax-AWS).

**Results:**

A total of 39 patients were included in the study. When both the proximal end of the fiberscope and ETT were simultaneously rotated by 90°, in the 6.5 group (n = 13), the distal end of the ETT rotated by 47.8 ± 1.5°. In the 7.0 °group (n = 13), the distal end of the ETT rotated by 45.5 ± 1.0°. In the 7.5 group (n = 13), the distal end of the ETT rotated by 39.9 ± 1.0°. When the proximal end of the fiberscope and ETT were rotated by 180°, in the 6.5 group, the distal end of the ETT rotated by 166.2 ± 2.5°. In the 7.0 group, the distal end of the ETT rotated by 145.7 ± 2.2°. In the 7.5 group, the distal end of the ETT rotated by 115.1 ± 2.0°. All rotation angles in the distal end of the ETT were significantly lower than those in both the proximal end of the fiberscope and ETT (p < 0.05). Rotating right by 180° was significantly different among the three groups (p < 0.05), although rotating right by 90° was not significantly different. Similar results were obtained for the left rotation.

**Conclusion:**

Simultaneous rotation by the proximal end of the ETT and fiberscope above the glottis for the nasal approach induced significant differences in the distal end of the ETT. The larger tube lagged by the resistance of the nasal passages during rotation. Therefore, the ETT does not rotate as much as the rotation angle.

**Trial registration:**

This prospective observational study was conducted after receiving approval from the Ethics Review Board of Kyushu University Hospital (Approval No. 30–447).

## Introduction

A nasal endotracheal tube (ETT) is routinely placed when full access to the oral cavity is required to facilitate surgical procedures [1–3]. Fiberoptic intubation, with limited mouth opening and/or neck mobility, is a useful technique in cases of difficult intubation [4–6].

However, advancing the ETT over the fiberoptic scope is difficult in 0–90% of patients when it hangs up on structures of the laryngeal inlet [3, 7, 8]. It may damage the larynx and/or the vocal cord, leading to a postoperative sore throat [9]. Moreover, tracheal intubation failed despite the insertion of the fiberoptic scope into the trachea because the ETT often impinged at the epiglottis or arytenoid cartilages [10–12].

Several maneuvers have been proposed to overcome this difficulty. The gap between the tip of the ETT and the fiberoptic scope can be reduced using a thicker fiberoptic scope and a thinner ETT [13–15]. Simultaneous rotation of the fiberoptic scope and ETT could lead to successful intubation by reducing impingement on the ETT [8, 10, 14–17]. However, the effect of various rotational angles on the fiberoptic scope and ETT is not supported by any evidence due to resistance to the passage of the ETT in the nasal cavity.

Therefore, this study aimed to assess the differential rotation of the ETT and fiberoptic scope above the glottis for the nasal approach.

## Methods

The Ethics Review Board of Kyushu University Hospital approved this observational prospective study (Approval No. 30–447). This study was registered with UMIN 000050118. The participants were patients (aged 20–80 years) with American Society of Anesthesiologists - physical status (ASA-PS): I-II who underwent oral and maxillofacial surgery under general anesthesia at Kyushu University Hospital. Patients with anticipated difficult airways such as narrow mouth opening, limited head and neck extension, upper respiratory tract disease, abnormal laryngeal structures and ASA III and IV were excluded from the study. Written informed consent was obtained from all the participants. This study was conducted in accordance with the guidelines of the Declaration of Helsinki.

Patients were nasotracheally intubated for oral and maxillofacial surgery. Three sizes of nasotracheal ETTs (Portex®, North Facing Nasal Soft-Seal Cuffed Polar Preformed Endotracheal Tube, Smiths Medical International, Hythe, UK) were used in this study. They were divided into three groups according to the size of ETTs; the ETT internal diameter (ID) 6.5 mm (6.5 group), ID 7.0 mm (7.0 group), and ID 7.5 mm (7.5 group). An ETT of appropriate size for nasotracheal intubation was selected based on the diameter of the trachea in the chest X-ray, weight and height [18]. However, when we encountered a narrow nasal cavity or passage through the nares for nasal intubation, the ETT was replaced by a smaller size. General anesthesia was induced with propofol, atropine, remifentanil, fentanyl, and rocuronium. Following loss of consciousness, we prepped the patient for intubation with the left nasal cotton swabs soaked with a 2% lidocaine and 1:200,000 epinephrine solution. Insertion through the left nostril was facilitated with a lubricated cuffed 6.5, 7.0, or 7.5 mm ID nasotracheal ETT. A lubricated fiberscope with a 4.8 mm outer diameter (Pentax, HOYA, Tokyo, Japan) was advanced through the left nostril by an experienced anesthesiologist. The distal end of the ETT and fiberoptic scope were positioned above the glottis at a 0° rotation after the ETT was introduced through the nasal cavity with the lower pathway into the throat. The head was in a neutral position.

**Fig. 1:**
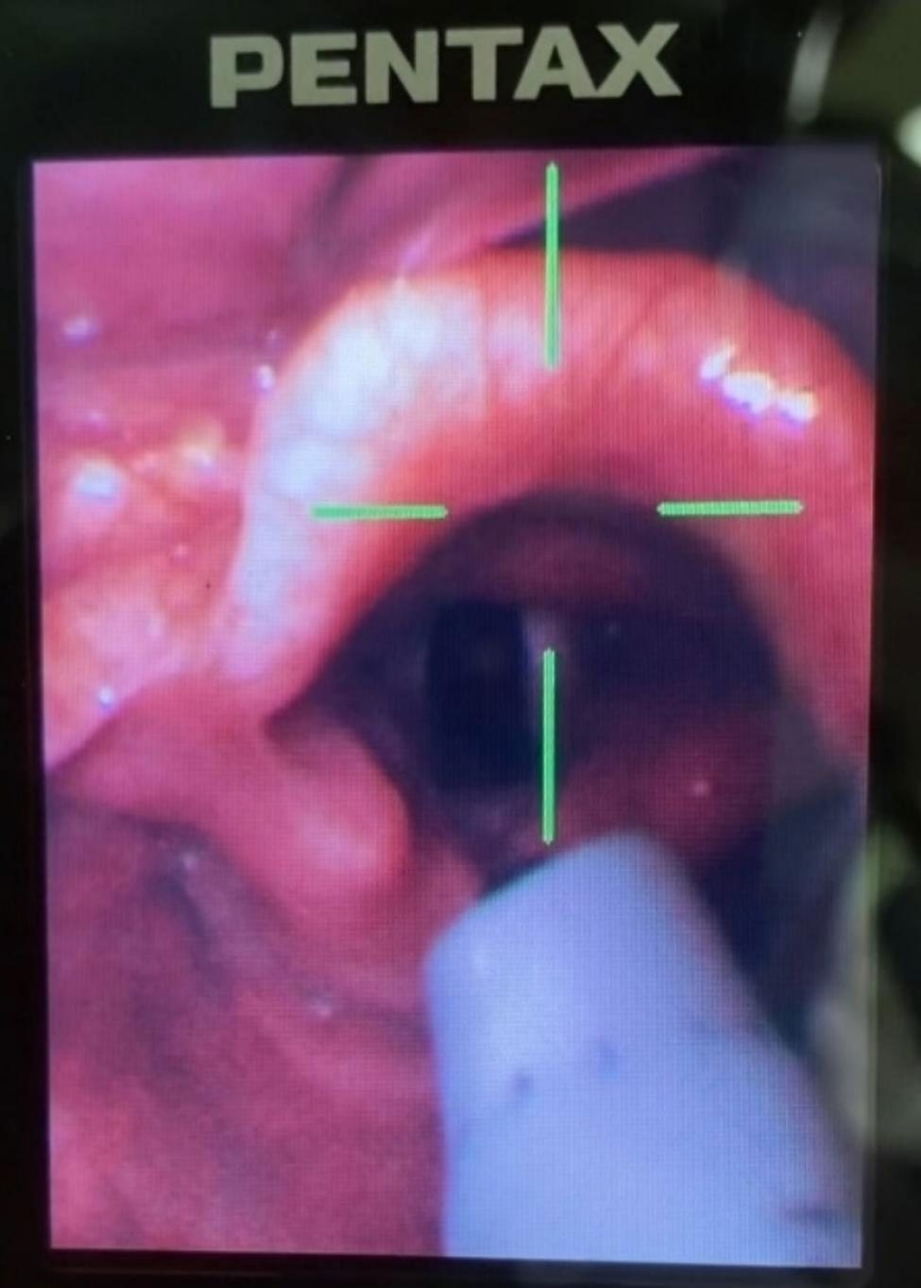
The distal end of fiberoptic scope and ETT were positioned above the glottis. The view of the laryngeal structures and rotation were monitored by Pentax-AWS (Pentax Corporation, Tokyo, Japan)

The fiberscope-ETT unit simultaneously rotated (90°, 180°) in both right (clockwise) and left (counterclockwise) directions in a full view of the laryngeal structures with chin lift and jaw thrust was monitored by Pentax-AWS (Pentax Corporation, Tokyo, Japan) (Fig. 1). The lines with the tip of the ETT were drawn at 2 mm intervals, and the distance traveled was calculated from the ratio of the circumference of a circle to its diameter. This study protocol was stopped immediately if SpO_2_ dropped until 90%.

Following the study protocol, intubation was performed, and confirmed by the presence of an end-tidal CO_2_ waveform and adequate breath sounds at lung auscultation. The ETT was fixed on the nares using tape. The ETT cuff was inflated by injecting air into the pilot balloon using a 10 mL syringe. The cuff pressure was measured by connecting the pilot balloon to a calibrated manometer for continuous intracuff pressure monitoring of 10–25 cmH_2_O. The choice of anesthetic maintenance was determined by each anesthesiologist. The procedure was performed as planned.

### Data analysis

All statistical analyses were performed using JMP® 15 (SAS Institute Inc., Cary, NC, USA). The sample size calculation was based on a previous study, obtaining α = 0.05 and a power of 80%. A total of 14 patients per group were included. In total, 42 trials were conducted. Values are expressed as the mean ± standard deviation (SD). Migrations at 90°and 180° with 0° among the groups and left-right rotation differences were compared using analysis of variance (ANOVA) with Steel-Dwass or Student’s t-test. Statistical significance was set at P < 0.05.

## Results

Three patients were excluded because of difficulties operating the fiberoptic scope under Pentax-AWS monitoring. A total of 39 patients met the inclusion criteria, with a mean ± SD age of 41.7 ± 16.8 years (range, 20–72 years); 18 were women (Table 1). No intraoperative respiratory complications were observed.

**Table 1:** Demographic data

	6.5 group (n = 13)	7.0 group (n = 13)	7.5 group (n = 13)
Gender (M/F)	0/13	8/5	13/0
Age (yrs)	45.2 ± 18.9	43.3 ± 14.9	37.4 ± 15.3
Height (cm)	154.8 ± 5.4	162.0 ± 3.8	171.2 ± 4.8
Weight (kg)	51.4 ± 4.3	58.5 ± 10.0	63.2 ± 8.6
ASA classification (1/2)	10/3	9/4	11/2

Values are means ± standard deviation or number.

The end of the fiberoptic scope was rotated to the desired position (90°, 180°) in all the cases. When the proximal end of the fiberoptic scope and ETT were simultaneously rotated by 90°, in the 6.5 group (n = 13), the distal end of the ETT rotated by 47.8 ± 1.5°. In the 7.0 °group (n = 13), the distal end of the ETT rotated by 45.5 ± 1.0°. In the 7.5 group (n = 13), the distal end of the ETT rotated by 39.9 ± 1.0°. When both the proximal end of the fiberoptic scope and ETT were rotated by 180°, in the 6.5 group, the distal end of the ETT rotated by 166.2 ± 2.5°. In the 7.0 group, the distal end of the ETT rotated by 145.7 ± 2.2°. In the 7.5 group, the distal end of the ETT rotated by 115.1 ± 2.0°.

All rotation angles of the distal end of the ETT were significantly less than those at the proximal side of the fiberoptic scope and ETT (p < 0.05) (Table 2). Rotating right by 180° was significantly different among the three groups (p < 0.05), although rotating right by 90° was not significantly different. In the left rotation, similar results were obtained, although no significant differences were observed between the left and right rotation differences (p > 0.05).

**Table 2:** The migrations of rotation of the distal end of endotracheal tube (ETT)

	6.5 group	7.0 group	7.5 group	P value
The migrations at right rotation at an angle of 90° (angle °)	47.8 ± 1.5	45.5 ± 1.0	39.9 ± 1.0	P = 0.32
The migrations at right rotation at an angle of 180° (angle °)	166.2 ± 2.5	145.7 ± 2.2	115.1 ± 2.0	P < 0.05
The migrations at left rotation at an angle of 90° (angle °)	46.9 ± 1.7	47.3 ± 0.8	39.0 ± 0.7	P = 0.26
The migrations at left rotation at an angle of 180° (angle °)	160.1 ± 3.2	144.8 ± 1.8	105.8 ± 1.7	P < 0.05

Each group was compared using analysis of variance (ANOVA) with Steel-Dwass test or Student t-test. Values are means ± standard deviation. The significant level was set at less than 5%.

Rotated right and left by 180° were significantly different among 3 groups (p < 0.05), although rotated right and left by 90° were not significantly different (p > 0.05).

All rotation angles in the distal end of ETT were significantly less than those of the proximal end of fiberoptic scope and the ETT (p < 0.05).

No significant difference was observed left and right rotation in each group (p > 0.05).

## Discussion

This study aimed to quantitatively assess the difference in the rotation of the ETT and fiberoptic scope above the glottis using the nasal approach. The findings of this study indicated that simultaneous rotation of both the proximal end of the fiberoptic scope and ETT could produce significant differences in the rotation angle with the distal end of the ETT. Furthermore, rotation with the larger ETT could result in greater resistance. However, no significant differences were observed between the left and right rotations (p > 0.05).

The primary reason for the differential rotation might be resistance to the passage of the ETT against rotation in the nasal cavity and the softness of the ETT materials [5]. The ETT is gripped by the walls of the nasal passage, resulting from deviation of the nasal septal spur or mucosal thickening of the turbinate [8]. Therefore, ETT twists can occur, to some extent, rather than rotate [17]. The proximal end of the fiberoptic scope and ETT might have been rotated to the desired position; however, the distal end of the ETT could not be rotated. We observed that a proximal rotational movement was not always translated into the distal part of the ETT because the ETT lagged behind the fiberscope [16, 19, 20]. Therefore, the ETT does not rotate as much as the rotation angle.

In this study, we used the chin lift with head tilt and jaw thrust maneuver to observe the airway during the rotation, which can reflect the physiological consequences of an increase in the pharyngeal airway cross-sectional area by the tongue base away from the posterior pharyngeal wall and actively moves the epiglottis away from the pharyngeal wall to provide more space in the pharynx [8, 11–13]. This airway-supporting maneuvers could be maintained correctly during the study [15].

Our study had two limitations. First, fiberoptic intubation can be difficult and requires operator skill, and the consequences of failure are significant. In this study, fiberoptic intubation was performed by an experienced anesthesiologist. Second, the technique of fiberoptic intubation is likely done while the patient is awake, so, this may lead to variant results from the current study.

## Conclusion

Simultaneous rotation by the proximal end of the ETT and fiberscope above the glottis for the nasal approach induced significant differences in the distal end of the ETT. The larger tube lagged by the resistance of the nasal passages during rotation. Therefore, the ETT does not rotate as much as the rotation angle.

## Data Availability

The datasets used and/or analyzed during the current study are available from the corresponding author on reasonable request.
